# Sex Differences in Sphingosine-1-Phosphate Levels Are Dependent on Ceramide Synthase 1 and Ceramidase in Lung Physiology and Tumor Conditions

**DOI:** 10.3390/ijms241310841

**Published:** 2023-06-29

**Authors:** Michela Terlizzi, Chiara Colarusso, Giusy Ferraro, Anna Falanga, Maria Chiara Monti, Pasquale Somma, Ilaria De Rosa, Luigi Panico, Aldo Pinto, Rosalinda Sorrentino

**Affiliations:** 1 Department of Pharmacy (DIFARMA), University of Salerno, 84084 Fisciano, Italy; ccolarusso@unisa.it (C.C.); gferraro@unisa.it (G.F.); afalanga@unisa.it (A.F.); mcmonti@unisa.it (M.C.M.); aldopinto952@gmail.com (A.P.); 2 Anatomy and Pathology Unit, Ospedale dei Colli, Azienda Ospedaliera di Rilievo Nazionale (AORN), “Monaldi”, 84131 Naples, Italy; somma.pasquale@tiscali.it (P.S.); ilaria.derosa@ospedalideicolli.it (I.D.R.); lupanico59@gmail.com (L.P.)

**Keywords:** sphingosine-1-phosphate (S1P), sex differences, S1P metabolism, S1PR3, lung cancer

## Abstract

Sex is a biological variable that can reflect clinical outcomes in terms of quality of life, therapy effectiveness, responsiveness and/or toxicity. Sphingosine-1-phosphate (S1P) is a lipidic mediator whose activity can be influenced by sex. To evaluate whether the S1P axis underlies sex ‘instructions’ in the lung during physiological and oncological lung conditions, sphingosine and S1P were quantified in the blood of healthy (H) volunteers, lung adenocarcinoma (ADK) and squamous cell carcinoma (SCC) patients of both sexes. S1P receptors and their metabolic enzymes were evaluated in the tissues. Circulating levels of S1P were similar among H female and male subjects and female SCC patients. Instead, male and female ADK patients had lower circulating S1P levels. S1P receptor 3 (S1PR3) was physiologically expressed in the lung, but it was overexpressed in male SCC, and female and male ADK, but not in female SCC patients, who showed a significantly reduced ceramide synthase 1 (CERS1) mRNA and an overexpression of the ceramidase (ASAH1) precursor in lung tumor tissues, compared to male SCC and both male and female ADK patients. These findings highlighted sex differences in S1P rheostat in pathological conditions, but not in physiological conditions, identifying S1P as a prognostic mediator depending on lung cancer histotype.

## 1. Introduction

Sphingosine-1-phosphate (S1P) is a multifunctional bioactive lipid and a critical regulator of many physiological and pathological processes. S1P is a metabolite of ceramide synthetized by two major mechanisms: the de novo and sphingomyelinase pathways. The de novo pathway is an anabolic route that begins with the condensation of the amino acid serine with palmitoyl-CoA, by means of the serine palmitoyl transferase (SPT), to form 3-ketodihydrosphinganine, which is then reduced into dihydrosphinganine by means of 3-ketodihydrosphingosine reductase (KDSR). The acylation of the dihydrosphinganine by the ceramide synthase 1–6 (CERS1–6) generates dihydroceramide. The introduction of a trans-4, 5 double bound in the dihydroceramide molecule by dihydroceramide desaturase 1 or 2 (DEGS1 and 2) leads to ceramide production. The second major mechanism of ceramide generation is a catabolic pathway that involves the activation of the sphingomyelinases 1, 2 or 3 (SMPD1, 2 and 3), which degrades a membrane sphingolipid, sphingomyelin, into phosphorylcholine and ceramide. The ceramide is converted by the ceramidases (ASAH1 and 2, ACER1, 2 and 3) into sphingosine, a substrate of two sphingosine kinases (SPHKs), SPHK I and SPHK II, which generate S1P [1].

S1P can exert intracellular functions or can be transported through the cell membrane in an inside-out mode by means of Spinster homolog 2 (SPNS2), a non-ATP-dependent organic ion transporter that is overexpressed in mammalian endothelial, immune [2], and epithelial cells [3], or by means of Mfsd2b, which is responsible for releasing S1P from erythrocytes and platelets [4]. Once released, S1P can bind to the extracellular G-coupled receptors (S1PR1–5) [5,6] and carry out countless biological activities [4,7,8,9]. S1P’s activities have been described as both physiological and pathological, as it can participate in chemotaxis, cell migration and proliferation, angiogenesis, heart chronotropism and inotropism, endothelial permeability, and blood vessel integrity and tone [10,11]. Among its pleiotropic activities, it has been demonstrated by others and by our group that S1P can be involved in lung inflammatory pathways [12,13,14,15,16,17,18]. In particular, the activation of TLR9 in lung epithelial cells can promote the synthesis of S1P, thereby increasing the levels of TNF-α [19] as well as cell proliferation [3] in both a TLR9- and S1PR3-dependent manner. As it was recently proved that the activation of the immune system and thus the ensuing inflammatory pathways are strictly related to sex, in that males and females differ in their immunological responses to foreign and self-antigens, suggesting that hormones can affect the immune response [20,21,22,23], it is likely that sex is a biological variable that can impact both lung physiology and pathologies. In accordance with this, it was shown that estrogens can stimulate S1P synthesis in females [24]. Instead, testosterone can limit S1P synthesis [25,26]. Moreover, while circulating estrogens from the ovary (mainly β-estradiol and estrone) are regulated by menstrual status and are much higher in premenopausal than in postmenopausal females, local sources of estrogen in tissues (which may not enter the circulation) are also important. The enzyme CYP19 (aromatase), which converts testosterone to estrone and β-estradiol, is present in the pulmonary microenvironment as a source of local estrogen production in both females and males [27]. In addition to expression in bronchial epithelial cells and lung carcinomas, aromatase is also expressed by pulmonary macrophages and other inflammatory cells in the lung [27,28]. Testosterone can be converted locally in the pulmonary microenvironment to estrogen, independent of the reproductive system, regulating S1P levels/signaling. Based on these findings, the aim of this study was to investigate the role of circulating S1P as a potential biomarker between females and males to discriminate between physiological status and pathological status as they correlate to lung homeostasis and malignant pathology. As a pathological condition of the lung, we considered non-small cell lung cancer (NSCLC), which accounts for 85% of all lung cancer cases [29]. It comprises two histological more common subtypes, adenocarcinoma (ADK) and squamous cell carcinoma (SCC). Epidemiological studies have revealed that ADK counts for 40–60% of cases, with a higher incidence in females, while SCC counts for 10–30% of cases in females versus 30–55% in males [30]. Although sex-related incidence varies according to the histotype, the absolute 5-year survival rate for NSCLC is 26%, with a higher median survival in females than males [31]. Therefore, in the era of sex-specific medicine, it is clear that discrimination between physiological status and the occurrence of pathologies is of significant clinical relevance.

## 2. Results

### 2.1. Circulating Levels of S1P in Physiological Conditions: A Comparison between Females and Males

**Figure 1:**
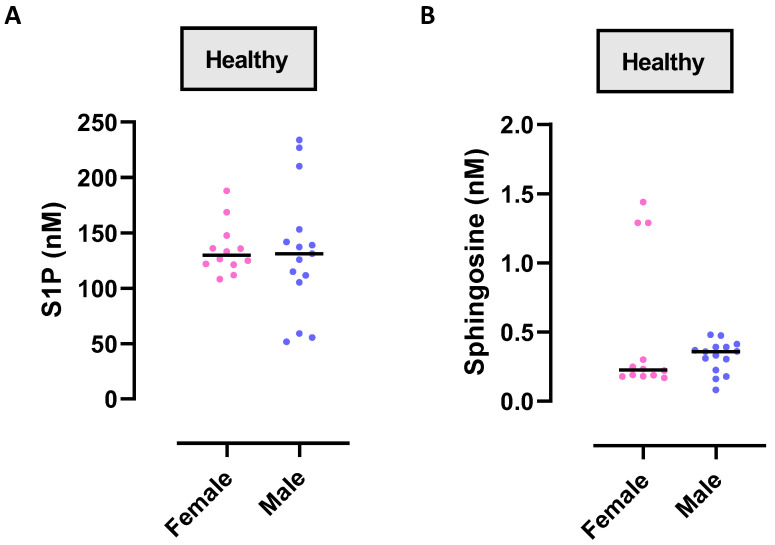
Circulating S1P and sphingosine in healthy subjects. S1P (**A**) and sphingosine (**B**) were quantified by means of LC-MS/MS in the plasma of healthy subjects: n = 12 female and n = 15 males. Data are represented as scatter dot plots indicating the median.

To evaluate any difference in S1P synthesis/levels according to the sex, we analyzed the plasma levels of S1P in healthy volunteers by using LC-MS/MS. We found that S1P plasma levels were similar in both females and males (Figure 1A; female S1P median: 129.97 nM vs. male S1P median: 131.06 nM). Similarly, circulating levels of sphingosine were not altered between females and males (Figure 1B; female sphingosine median: 0.227 nM vs. male sphingosine median: 0.359 nM), although they were much lower (female S1P median: 129.97 nM vs. female sphingosine median: 0.227 nM, *p* < 0.0001; male S1P median: 131.06 nM vs. male sphingosine median: 0.359 nM, *p* < 0.0001) than S1P.

### 2.2. Circulating S1P Levels in NSCLC Patients: Comparison between Females and Males

To understand the role of S1P in sex differences associated with lung neoplasia, we analyzed plasma levels of S1P in NSCLC patients, both ADK and SCC, as well as females and males.

First, we found that circulating levels of S1P were significantly reduced in ADK patients compared to healthy (H) subjects (Supplementary Figure S1A; ADK median: 86.63 nM [67.69–93.87 range] vs. H median: 131.1 nM [115–141.8 range]; *p* < 0.0001), while no significant differences were found between SCC patients and H volunteers (Supplementary Figure S1A; SCC median: 108.1 nM [81.06–129 range] vs. H median: 131.1 nM [115–141.8 range]; *p* = 0.0638). Sphingosine levels showed a similar trend, although its levels were much lower than S1P (H S1P median: 131.059 nM vs. H sphingosine median: 0.3215 nM, *p* < 0.0001; ADK S1P median: 86.629 nM vs. ADK sphingosine median: 0.251 nM, *p* < 0.0001; SCC S1P median: 108.148 nM vs. SCC sphingosine median: 0.522 nM, *p* < 0.0001) (Supplementary Figure S1B). Because we already demonstrated that lung cancer epithelial cells synthetize and release high levels of S1P [19], and that circulating S1P acts on its receptors both on lung cancer tissues [3,19] and circulating cells [32], these data may imply either that S1P from the plasma is driven toward receptors in the case of ADK patients, or that a compromised or non-relevant S1P metabolism/synthesis occurs in the SCC histotype.

**Figure 2:**
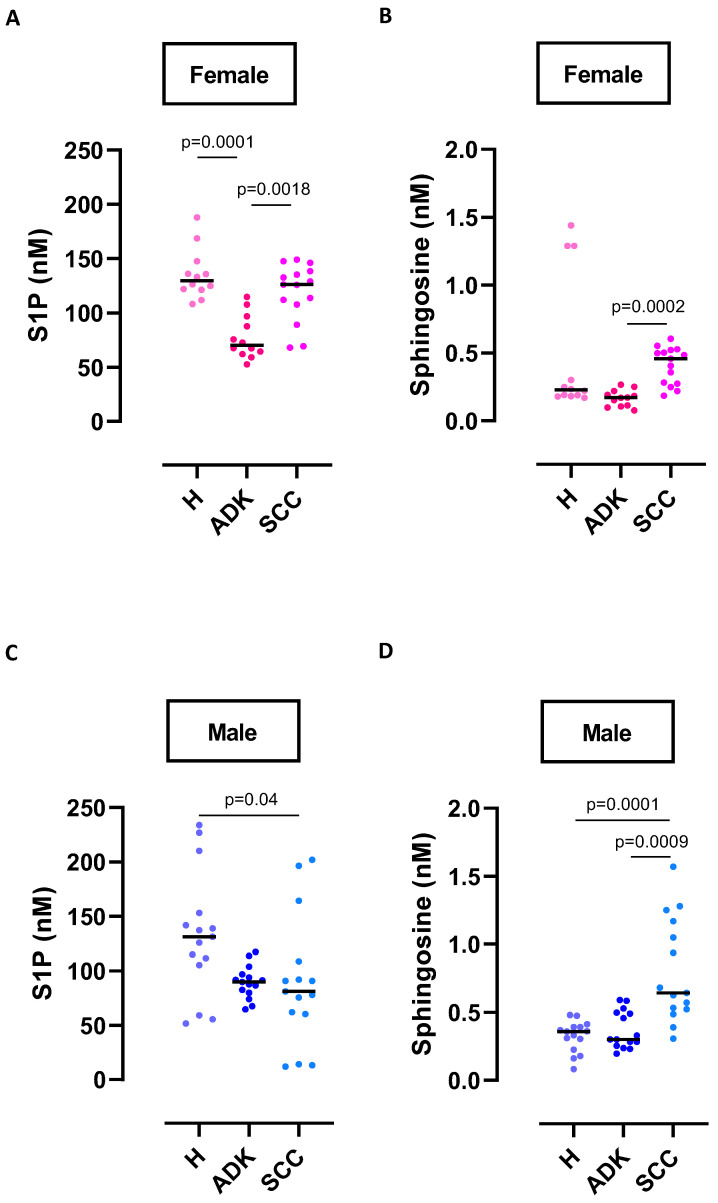
Circulating S1P and sphingosine in lung cancer patients. S1P and sphingosine were quantified in the plasma of ADK and SCC patients, both females and males, by means of LC-MS/MS. (**A**) Female ADK (n = 12) showed lower levels of S1P compared to H female (n = 12) and female SCC (n = 15). (**C**) H males (n = 15) showed higher levels of circulating S1P compared to both males with ADK (n = 15) and SCC (n = 15). Additionally, sphingosine levels were evaluated in the same groups (**B**,**D**). Data are represented as scatter dot plots indicating the median. Statistical differences were assessed by means of one-way ANOVA followed by Dunn’s post hoc test. H: healthy; ADK: lung adenocarcinoma; SCC: lung squamous cell carcinoma.

To understand any sex difference, NSCLC patients were stratified according to the sex and tumor histotype. We found that circulating levels of S1P were significantly reduced in female ADK patients compared to female H subjects (Figure 2A; female ADK median: 70.13 nM [61.98–96.80 range] vs. female H median: 129.7 nM [121.2–147.6 range], *p* = 0.0001). Instead, SCC female patients had similar levels of plasma S1P to female H subjects (Figure 2A; female SCC median: 126 [107.7–138.3 range] vs. H female median: 129.7 nM [121.2–147.6 range]). Sphingosine levels showed a similar trend, although its levels were much lower than S1P (H S1P median: 129.7 nM vs. H sphingosine median: 0.227 nM, *p* < 0.0001; ADK S1P median: 70.13 nM vs. ADK sphingosine median: 0.1705 nM, *p* < 0.0001; SCC S1P median: 126 nM vs. SCC sphingosine median: 0.457 nM, *p* < 0.0001) (Figure 2B). Nevertheless, it should be noted that sphingosine levels in female SCC were higher than in H and ADK, although at lower concentrations.

In contrast, male SCC patients had significantly lower plasma levels of S1P than male H subjects (Figure 2C; male SCC median: 81.06 nM [60.31–108.6 range] vs. male H median: 131.1 nM [105.3–153.2 range]); S1P levels in male ADK patients were also lower compared to male H subjects, although not statistically significant (Figure 2C; male ADK median: 89.83 nM [79.81–96.80 range] vs. male H median: 131.1 nM [105.3–153.2 range]). Instead, sphingosine levels were higher in male SCC compared to male ADK patients and male H subjects (Figure 2D), although much lower than S1P (H S1P median: 131.1 nM vs. H sphingosine median: 0.359 nM, *p* < 0.0001; ADK S1P median: 89.83 nM vs. ADK sphingosine median: 0.3 nM, *p* < 0.0001; SCC S1P median: 81.06 nM vs. SCC sphingosine median: 0.643 nM, *p* < 0.0001). To rule out the different levels of plasma S1P being related to its dephosphorylation by means of sphingosine-1-phosphate phosphatases (SGPP1 and SGPP2, enzymes that convert S1P into sphingosine), or its degradation by means of sphingosine-1-phosphate lyase (SGPL1, an enzyme that degrades S1P into hexadecenal and phosphoethanolamine), we evaluated these enzyme transcript levels in lung cancer tissues. We did not find differences in SGPP1 between females and males in both ADK (Supplementary Figure S1C) and SCC (Supplementary Figure S1D) patients; meanwhile, SGPP2 was increased in female ADK compared to male ADK patients (Supplementary Figure S1E), but it did not differ between female and male SCC patients (Supplementary Figure S1F). SGPL1 did not differ between females and males in both ADK (Supplementary Figure S1G) and SCC (Supplementary Figure S1H) patients.

Altogether, these results show reduced levels of plasma S1P in both female and male ADK and male SCC patients, implying the higher biological activity of S1P in ADK (both females and males) and SCC males. Instead, female SCC patients seemed to have a dysfunctional S1P utilization if compared to male and female ADK; however, if compared to H subjects, they had similar levels of circulating S1P. Therefore, the utilization of S1P in female SCC was similar to that in H subjects, highlighting that the S1P axis is more active in ADK patients.

### 2.3. S1PR3 Expression in Non-Cancerous and Cancerous Lung Tissues of ADK and SCC Patients: Comparison between Females and Males

S1P performs its biological functions through interaction with its five G protein-coupled receptors (S1PR1–5) [6,33]. Because in our previous data we observed that circulating levels of S1P were reduced in ADK (male and females) and SCC (solely males), we assumed that S1P was driven toward its tissue receptors. Therefore, we evaluated the expression of S1P receptors in non-cancerous and cancerous lung tissues of female and male patients.

In these series of experiments, because it was not possible to obtain lung tissues from healthy subjects, for obvious ethical reasons, we used non-cancerous tissues that did not have tumor lesions, being anatomically derived far away from the tumor mass (as assessed by the clinical pathologist); these were obtained from ADK or SCC patients and used as reference controls.

We found that lung cancer tissues expressed mainly S1PR1, S1PR2 and S1PR3 (Supplementary Figure S2A), whereas S1PR4 and S1PR5 were undetectable, probably because they are highly restricted to distinct cell types and tissues, such as the lymphoid tissues, brain, and spleen [34]. Among the detectable receptors, we found that S1PR3 (median: 1.742 [1.259–2.221 range]) was the most expressed, and that its expression was higher compared to S1PR1 (median: 0.9204 [0.6533–1.875]) and S1PR2 (median: 1.033 [0.8426–1.616]) (Supplementary Figure S2B; S1PR3 vs. S1PR1 *p* = 0.036; S1PR3 vs. S1PR2 *p* = 0.1096).

**Figure 3:**
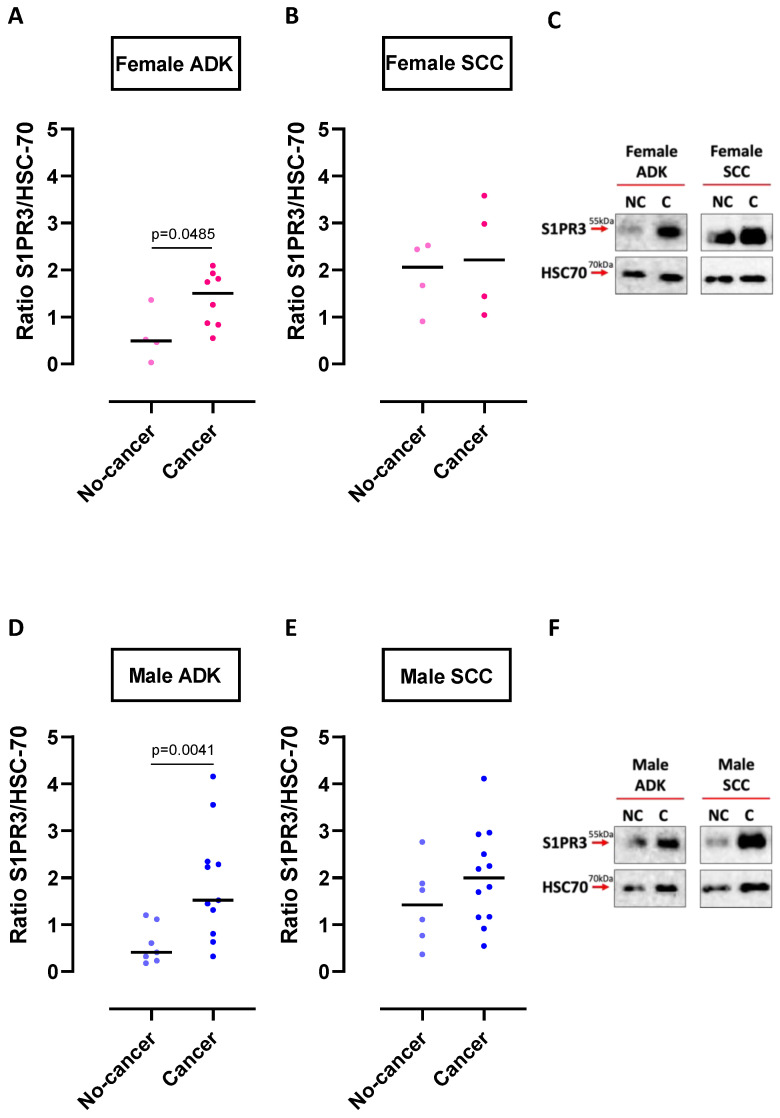
S1PR3 expression in lung tissues of ADK and SCC patients. Western blotting analysis showed that female (**A**) and male (**D**) ADK patients had higher levels of S1PR3 (55 kDa) in cancerous tissue (cancer, female n = 8; male n = 11) compared to the respective non-cancerous tissues (non-cancer, female n = 4; male n = 7). S1PR3 expression was similar in non-cancerous (non-cancer, female n = 4; male n = 6) and cancerous (cancer, female n = 4; male n = 12) tissues of SCC patients, regardless of sex (**B**,**E**). S1PR3 detection was performed on the same blot when evaluating ADK or SCC patients. Representative blot of S1PR3 expression in non-cancerous (NC) and cancerous (C) tissues of female (**C**) and male (**F**) ADK and SCC patients. HSC70 (70 kDa) was used as the loading control. A quantitative analysis was performed using ImageJ software (1.53a http://imagej.nih.gov/ij, accessed on 1 October 2022, NIH, Bethesda, MD, USA). Data are represented as scatter dot plots indicating the median. Statistical differences were assessed by means of a two-tailed Mann–Whitney U test. S1PR3: sphingosine-1-phosphate receptor 3; HSC70: Heat shock cognate protein 70; ADK: lung adenocarcinoma; SCC: lung squamous cell carcinoma.

Stratifying patients by tumor histotype, we found that S1PR3 was more expressed in cancerous than non-cancerous lung tissues of ADK patients (Supplementary Figure S2C,E). Instead, we did not observe any difference between non-cancerous and cancerous lung tissues obtained from SCC patients (Supplementary Figure S2D,E). However, it has to be pointed out that the non-cancerous tissues of SCC patients showed higher expression (median 1.8 [0.9045–2.549 range]) of S1PR3 than those of ADK patients (median 0.46 [0.1745–1.203 range]), a difference that was statistically significant (*p* = 0.0021) (Supplementary Figure S2D, light green dots vs. Supplementary Figure S2C, yellow dots). In addition, stratifying patients by tumor histotype and according to the sex, we found that S1PR3 was more expressed in cancerous than non-cancerous lung tissues of female ADK patients (Figure 3A,C). Instead, we did not observe any difference between non-cancerous and cancerous lung tissues obtained from SCC females (Figure 3B,C). Similarly, male ADK tissues had higher levels of S1PR3 than non-cancerous tissues (Figure 3D,F). Both non-cancerous and cancerous tissues obtained from male SCC patients showed similar levels of S1PR3 (Figure 3E,F). Representative blots are in Figure 3C for female and Figure 3F for male patients.

These data imply that S1PR3 is more highly expressed in cancerous tissues than normal lungs. Interestingly, analyzing the circulating levels of S1P and S1PR3 in the tissues, a higher S1PR3 expression corresponded to a reduction in S1P in the blood of both sexes in ADK and male SCC patients, but not in female SCC (Table 1), implying a dysfunctional usage of S1P in this latter group. Thus, we were prompted to investigate the expression of mRNA of the specific enzymes involved in S1P metabolism.

### 2.4. Evaluation of S1P Metabolic Enzymes in Non-Cancerous and Cancerous Lung Tissues of Female and Male SCC Patients

**Figure 4:**
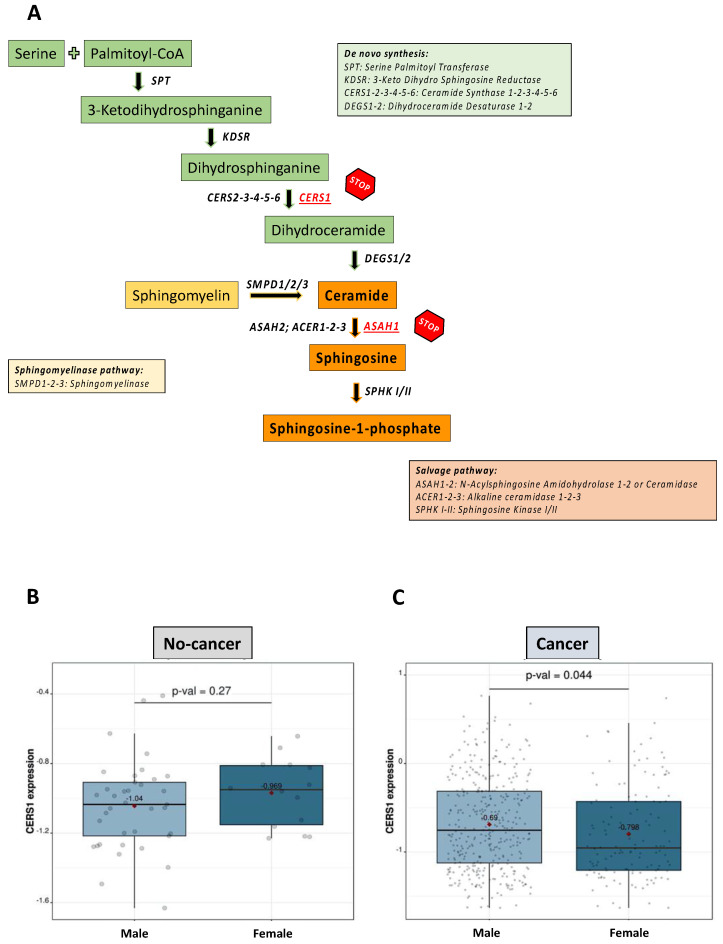
Ceramide synthase 1 (CERS1) is the limiting enzyme of S1P de novo synthesis in female SCC patients. (**A**) S1P biosynthetic pathway. (**B**) Data from RNAseq database TCGA_LUSC_2016 showed that CERS1 was equally expressed in non-cancerous tissues of male (n = 37) and female (n = 14) patients, while it was reduced in the cancerous lung tissues of female (n = 130), compared to male (n = 371) SCC patients (**C**). A comparative analysis of transcripts with default parameters was performed using the Lung Cancer Explorer (LCE) tool.

S1P can be synthesized through the de novo pathway, starting from serine and palmitoyl-CoA condensation (Figure 4A, green squares), or can derive from the hydrolysis of complex lipids, mainly sphingomyelin (Figure 4A, yellow squares). To understand whether S1P lung tissue synthesis processes changed in various physiological conditions and in NSCLC in both males and females, we took advantage of an RNAseq database of SCC (TCGA_LUSC_2016). We considered only SCC, as it was the tumor histotype that had an anomalous signaling of S1P compared to ADK. No differences were observed in S1P metabolism in ADK patients.

We did not find differences in serine palmitoyl-transferase (SPT) subunits (SPTLC1, SPTLC2 and SPTSSA; Supplementary Figure S3A–F; A. Non-cancer SPTLC1 mean expression: male 1.031 and female 1.029 *p* = 0.47; B. Cancer SPTLC1 mean expression: male 1.04 and female 1.028 *p* = 0.32; C. Non-cancer SPTLC2 mean expression: male 1.015 and female 0.984 *p* = 0.22; D. Cancer SPTLC2 mean expression: male 0.904 and female 0.885 *p* = 0.27; E. Non-cancer SPTSSA mean expression: male 1.126 and female 1.186 *p* = 0.16; F. Cancer SPTSSA mean expression: male 1.094 and female 1.059 *p* = 0.15) and 3-ketodihydrosphingosine reductase (KDSR) (Supplementary Figure S3G,H; G. Non-cancer KDSR mean expression: male 1.034 and female 1.043 *p* = 0.65; H. Cancer KDSR mean expression: male 0.983 and female 0.999 *p* = 0.19), the enzymes involved in the first two steps of the de novo synthesis of S1P, between males and females, both in non-cancerous and cancerous lung tissues. Surprisingly, the ceramide synthase 1 (CERS1), the key enzyme for ceramide synthesis, was equally expressed in non-cancerous lung tissues of males and females (Figure 4B; non-cancer CERS1 mean expression: male −1.04 and female −0.969 *p* = 0.27), but its transcription was significantly decreased in females with SCC compared to males (Figure 4C; Cancer CERS1 mean expression: male −0.69 and female −0.798 *p* = 0.044). No differences were found in the other ceramide synthase isoforms, CERS2, 3, 4, 5 and 6, between males and females, in both non-cancerous and cancerous lung SCC tissues (Supplementary Figure S4A–F; A. Non-cancer CERS2 mean expression: male 1.472 and female 1.478 *p* = 0.72; B. Cancer CERS2 mean expression: male 1.329 and female 1.338 *p* = 0.44; C. Non-cancer CERS3 mean expression: male −1.35 and female −1.42 *p* = 0.18; D. Cancer CERS3 mean expression: male 0.316 and female 0.198 *p* = 0.13; E. Non-cancer CERS4 mean expression: male 0.782 and female 0.803 *p* = 0.4; F. Cancer CERS4 mean expression: male 0.723 and female 0.684 *p* = 0.16; and Supplementary Figure S5A–D; A. Non-cancer CERS5 mean expression: male 0.735 and female 0.732 *p* = 0.83; B. Cancer CERS5 mean expression: male 0.821 and female 0.817 *p* = 0.72; C. Non-cancer CERS6 mean expression: male 0.612 and female 0.609 *p* = 0.95; D. Cancer CERS6 mean expression: male 0.945 and female 0.958 *p* = 0.53).

The other enzymes involved in the de novo S1P synthesis, dihydroceramide desaturase 1 and 2 (DEGS1 and DEGS2), did not differ between the groups considered (non-cancerous male vs. non-cancerous female and cancerous male vs. cancerous female; Supplementary Figure S6A–D; A. Non-cancer DEGS1 mean expression: male 1.073 and female 1.094 *p* = 0.32; B. Cancer DEGS1 mean expression: male 1.06 and female 1.052 *p* = 0.63; C. Non-cancer DEGS2 mean expression: male −0.0339 and female 0.047 *p* = 0.31; D. Cancer DEGS2 mean expression: male −0.224 and female −0.215 *p* = 0.84).

In addition to the de novo synthesis, S1P can also derive from the hydrolysis of sphingomyelin, which is mediated by acid and neutral sphingomyelinase (SMPD1, 2 and 3). The transcripts expression of these enzymes did not differ in non-cancerous tissues of both males and females (Supplementary Figure S7A,C,E; A. Non-cancer SMPD1 mean expression: male 0.913 and female 0.893 *p* = 0.45; C. Non-cancer SMPD2 mean expression: male 0.356 and female 0.333 *p* = 0.46; E. Non-cancer SMPD3 mean expression: male 0.208 and female 0.187 *p* = 0.75). Surprisingly, SMPD1 and 3 were overexpressed in cancerous tissues of female SCC compared to male SCC (Supplementary Figure S7B,F; B. Cancer SMPD1 mean expression: male 0.634 and female 0.692 *p* = 0.00047; F. Cancer SMPD3 mean expression: male −0.164 and female −0.0811 *p* = 0.0081), implying that sphingomyelin hydrolysis was not blocked in female SCC. No differences were noted in the SMPD2 transcripts’ expression in cancerous lung tissues of males and females (Supplementary Figure S7D; Cancer SMPD2 mean expression: male 0.388 and female 0.381 *p* = 0.65).

Altogether, these data show that CERS1 is the limiting enzyme in the de novo synthesis of S1P in lung tumor tissues of female SCC patients, and that the sphingomyelinase pathway could overcome this dysfunction, promoting ceramide synthesis, the precursor of S1P, through the hydrolysis of sphingomyelin.

### 2.5. Ceramidase (ASAH1) Expression in Non-Cancerous and Cancerous Lung Tissues of Female and Male SCC Patients

**Figure 5:**
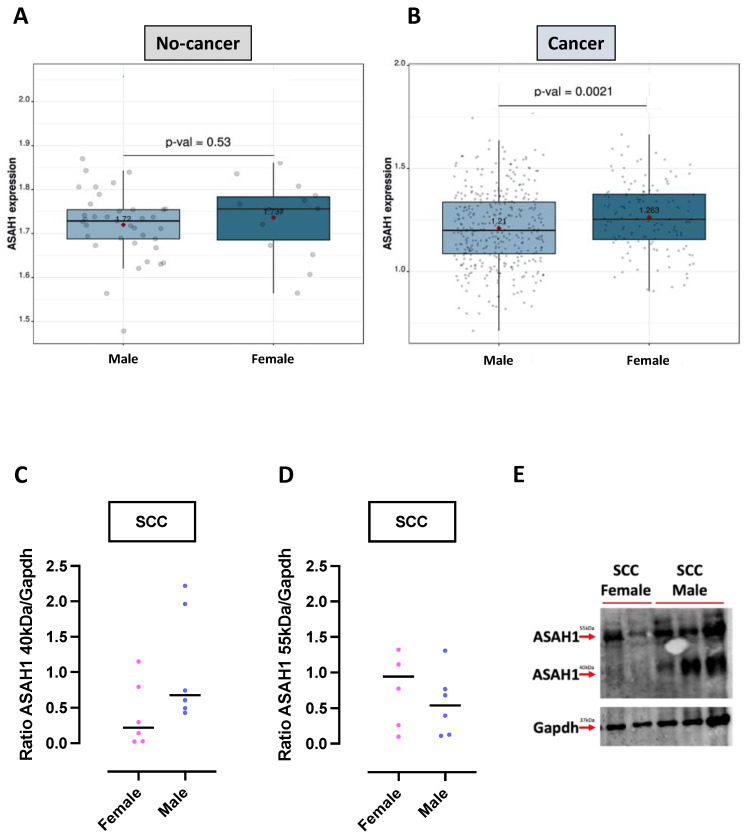
Ceramidase (ASAH1) is inactive in the lung tissues of female SCC patients. Data from RNAseq database TCGA_LUSC_2016 showed that ASAH1 was equally expressed in non-cancerous tissues of male (n = 37) and female (n = 14) (**A**), while it was increased in the cancerous lung tissues of female (n = 130), compared to male (n = 371) SCC patients (**B**). A comparative analysis of transcripts with default parameters was performed using the Lung Cancer Explorer (LCE) tool. (**C**) A Western blotting analysis showed that ASAH1 was over-expressed in its active form (40 kDa) in cancerous tissues of male compared to female SCC (**C**,**E**), while its precursor (55 kDa) was overexpressed in female compared to male SCC patients (**D**,**E**). Gapdh (35 kDa) was used as a loading control. The quantitative analysis was performed using ImageJ software (NIH, Bethesda, MD, USA). Data are represented as scatter dot plots indicating the median. SCC: lung squamous cell carcinoma; Gapdh: glyceraldehyde-3-phosphate dehydrogenase.

The synthesis of ceramides into sphingosine and S1P is prompted by the activation of the ceramidases (ASAH1 and 2, ACER1, 2 and 3) (Figure 4A, orange squares). ASAH1 is the most studied ceramidase; we have demonstrated its involvement in lung cancer [3,18,19,32]. According to the RNAseq database (TCGA_LUSC_2016), male and female non-cancerous lung tissues expressed similar levels of mRNA ASAH1 (Figure 5A; male mean expression: 1.72 vs. female mean: 1.737; *p* = 0.53). Instead, cancerous lung tissues of female SCC over-expressed mRNA ASAH1 compared to cancerous tissues of males (Figure 5B; male mean expression: 1.21 vs. female mean: 1.263; *p* = 0.0021). Since this analysis was limited by not discriminating between the active form and the inactive precursor of the enzyme, we performed a Western blotting analysis. We found that male SCC over-expressed the ASAH1 protein in its active form (40 kDa) compared to female SCC (Figure 5C,E; female median: 0.2189 vs. male median: 0.6739). Instead, female SCC patients showed higher levels of the ASAH1 protein in its inactive form (55 kDa) than male SCC groups (Figure 5D,E; female median: 0.9422 vs. male median: 0.5374).

Moreover, no statistical differences in mRNA were observed for SPHK I (Supplementary Figure S8A,B; A. Non-cancer SPHK I mean expression: male 0.225 and female 0.238 *p* = 0.9; B. Cancer SPHK I mean expression: male 0.661 and female 0.631 *p* = 0.31;) and SPHK II (Supplementary Figure S8C,D; C. Non-cancer SPHK II mean expression: male 0.407 and female 0.378 *p* = 0.45; D. Cancer SPHK II mean expression: male 0.462 and female 0.484 *p* = 0.15), an enzyme phosphorylating sphingosine in S1P, in both male and female non-cancerous and cancerous lung tissues.

In conclusion, while S1P synthesis is not altered during physiological conditions, females SCC patients, rather than male SCC or female/male ADK patients, have more inactive ASAH1 than its active form (female SCC inactive ASAH1 median: 0.9422 vs. female SCC active ASAH1 median: 0.2189). This may, on the one hand, be explained by differential physio-pathological alterations between SCC and ADK; on the other hand, it is likely that higher plasma S1P in female SCC patients may derive from cellular sources other than lung tissues.

## 3. Discussion

In this study, we found the following:

1. S1P circulating levels are not different between males and females during physiological conditions;
2. S1P metabolic pathways are not altered in males and females in physiological states;
3. Circulating S1P levels are higher in physiological conditions compared to pathological neoplastic conditions (as in the case of NSCLC);
4. Alteration of the ceramide/S1P axis makes the difference between male and female SCC patients, as well as SCC versus ADK patients;
5. Female SCCs show an altered metabolism of S1P due to an inactive ASAH1, which most likely does not need to be activated due to the absence of the substrate derived from CERS1, which mRNA is downregulated.

Epidemiological studies have widely reported that there are clear differences in survival rates for males and females after diagnosis of NSCLC [31]. The median survival for females is higher than for males diagnosed with NSCLC. If we focus on lung SCC, it is well known that this type of cancer has a very poor prognosis, and although there is a lower incidence in females, the survival rate is better for females (probably because they are more responsive to therapies) [30]. In this regard, we may speculate that the alteration of the ceramide/S1P axis may be of benefit for female SCC patients, who survive longer than male SCC patients, as well as male and female ADK patients, who instead have higher activity of S1P. The lungs of female SCC compared to male SCC patients have higher levels of inactive ASAH1, which represents a limiting factor for S1P generation, either via the de novo or the sphingomyelin-dependent pathway. Nevertheless, plasma levels of S1P in female SCC patients are not reduced, implying either that other cellular sources maybe responsible for its release, or that it does not prompt tissue signaling. Instead, the inactivation of ASAH1 and no change in S1PR3 expression in lung tissues correlated to higher S1P levels in the blood of female SCC patients; this result leads us to suppose that it is more likely that S1P does not exert tissue signaling. This could be a reason for the higher survival rate in female SCC. On the other hand, though, these data further support our previous studies, wherein we described S1P as a tumorigenic driver in ADK (both sexes) [3,18,19,32].

Furthermore, in our previous studies, we have shown that circulating S1P is able to amplify lung cancer-associated inflammatory processes through the interaction of S1P with S1PR3 both in circulating peripheral blood mononuclear cells (PBMCs) [32] and in lung cancer epithelial cells [19]. Therefore, lower tissue S1P activity in females with SCC, as well as in the physiological condition, could protect this group of patients from the deleterious effects associated with the exacerbation of inflammation. From our data, however, we determined that females with SCC express S1P receptors (S1PR3) in cancerous tissues in a manner similar to the other groups (males with SCC, and males and females with ADK). Therefore, although S1PR3 is over-expressed, this receptor could be dysfunctional or inappropriately activated in this group, given the low tissue levels of S1P in female SCC patients due to the blockade of CERS1 and ASAH1. This point needs further clarification, and is a limitation of the current study. Nevertheless, the lower levels of CERS1 and ASAH1 activity in female SCC patients led us to think that tissue S1P is underproduced, thus implying the lower activity of the S1PR3. Another limitation of this study that needs future elucidation is the role of S1P when released by peripheral blood cells in a pathological condition, as in the case of lung SCC.

In conclusion, our findings highlight sex differences in S1P rheostat in pathological but not in physiological conditions. These data could identify S1P as a mediator to highlight sex differences related to prognosis and/or clinical outcomes based on the tumor histotype.

## 4. Materials and Methods

### 4.1. Human Samples

Healthy (H) volunteers and non-small cell lung cancer (NSCLC) patients were recruited at the “Monaldi-Azienda Ospedaliera (AORN)-Ospedale dei Colli” Hospital in Naples, Italy, in accordance with the review board that approved the project and the patients’ informed consent. The experimental protocol was performed in accordance with the guidelines and regulations provided by the ethical committee of the hospital (protocol n. 1254/2014). The H volunteers’ group (n = 27) did not have any pathologies or hematological alterations. The NSCLC patients’ group (n = 57) consisted of adenocarcinoma (ADK, n = 27) patients, of whom n = 12 females and n = 15 males, and squamous cell carcinoma (SCC, n = 30) patients, of whom n = 15 females and n = 15 males (Supplementary Tables S1 and S2). The age of the enrolled volunteers and patients had a mean of 60 ± 15 years old. Blood samples were collected from H, ADK and SCC patients in polypropylene tubes containing EDTA. Within 24 h from the time of the withdrawal, blood was centrifuged at 1200 rpm for 10 min, and platelet-rich plasma (PRP) was obtained. A subsequent centrifuge at 3800 rpm was performed for 10 min in order to eliminate platelets (platelet-poor plasma, PPP), and the plasma was stored at −80 °C until needed for S1P quantification, as already reported [35]. Lung tissues were collected from ADK and SCC patients during surgery; these comprised a portion of tissue from the tumor mass (here identified as ‘cancerous’), and a portion from a distal part of the lung (not affected by the tumor lesion) of the same patient (here identified as ‘non-cancerous’), as assessed by the clinical pathologist, and as already reported [36]. Lung tissues were used to evaluate the expression of S1PR1, 2 and 3 and ceramidase, as described below.

### 4.2. Sphingosine and S1P Extraction from Human Plasma

A volume of 700 µL MeOH was added to 100 µL of plasma. Samples were stirred for 1 h at room temperature in a thermomixer under shaking (300 rpm), and centrifuged at 10,000× *g* for 5 min. The supernatant was transferred to a new centrifuge tube and evaporated to dryness at 40 °C in the vacuum rotator. Samples were reconstituted in 25 µL MeOH, vortexed for 5 min, sonicated for 10 min and centrifuged for 10 min at 10,000× *g*, and 5 µL was loaded on the UPLC-MSMS system.

### 4.3. LC-MS/MS Analysis

Sphingosine and S1P were quantified by liquid chromatography coupled to tandem mass spectrometry detection (LC-MS/MS) on Shimadzu LC-20A and Auto Sampler systems and a QTRAP 6500 instrument from AB-Sciex. A C18 chromatographic column (Kinetex C18, 50 × 2.1 mm, 5 µm, Phenomenex, Torrance, CA, USA) was used for chromatographic separation. The mobile phase was composed of water 0.1% formic acid (mobile phase A) and methanol 0.1% formic acid (mobile phase B). The flow rate was set at 400 µL/min. The mass spectrometer was set in the positive ion mode (ESI+) with an electrospray voltage of 5500 V at a heated capillary temperature of 400 °C. A multiple reaction monitoring (MRM) mode and Analyst 1.6.2 software was used. Sphingosine was analyzed using a mass transition of 300 m/z to 282 m/z, and it was observed at the rt (retention time) of 6.30 min. S1P was analyzed with a mass transition of 380 m/z to 264 m/z, and it was observed at the rt of 7.56 min. Nitrogen was used as the air curtain gas (20 psi), atomizing gas (30 psi), auxiliary gas (60 psi), and collision gas (4 psi). The dwell time was 100 ms, DP was 74 v, EP was 10 v, CE was 22 v and CXP was 15 v. For quantitative analysis, a standard curve of sphingosine (#S7049; Sigma-Aldrich, Merck Life Science S.r.l., Milan, Italy) and S1P (#S9666; Sigma-Aldrich, Merck Life Science S.r.l., Milan, Italy) (658.8, 263.5, 65.88, 26.35 and 6.58 nM) was run.

### 4.4. Western Blotting Analysis

The expression of S1PR1 (44 kDa; Bioss Antibodies; Woburn, MA, USA; #bs-7112R), S1PR2 (40–50 kDa; Fine Test; Wuhan, China; #FNab07569), S1PR3 (55–70 kDa; Alomone labs; Jerusalem, Israel; #ASR-013) and of ceramidase (active form: 40 kDa; precursor form: 55 kDa; N-acylsphingosine amidohydrolase 1, ASAH1; Elabscience; Houston, TX, USA; #E-AB-10959) was evaluated in ADK and SCC tissues. Glyceraldehyde-3-phosphate dehydrogenase (37 kDa; GAPDH; OriGene Technologies, Rockville, MD, USA; #TA890003) or heat shock cognate protein 70 (70 kDa; HSC-70; OriGene Technologies, Rockville, MD, USA; #TA332519) were used as the loading control. Data were analyzed using ImageJ software 1.53a. http://imagej.nih.gov/ij, accessed on 1 October 2022 (NIH, Bethesda, MD, USA). 

### 4.5. Analysis of Transcripts Expression

The Lung Cancer Explorer (LCE) online tool was applied (https://lce.biohpc.swmed.edu/lungcancer/) to evaluate S1P metabolism-associated transcripts’ expression in the lung tissues of ADK and SCC patients. Specifically, TCGA_LUAD_2016, a lung ADK database consisting of 575 total samples (58 normal samples, including 24 males and 34 females, and 517 tumor samples, including 240 males and 277 females), and TCGA_LUSC_2016, a lung SCC database consisting of 552 total samples (51 normal samples, including 37 males and 14 females, and 501 tumor samples, including 371 males and 130 females), were evaluated. A comparative analysis of transcripts with default parameters was performed using the LCE tool.

### 4.6. Statistical Analysis

Data are reported as median and represented as scatter dot plots. Statistical differences were assessed with a one-way ANOVA followed by Dunn’s post hoc test or a two-tailed Mann–Whitney U test, where appropriate. *p* values less than 0.05 were considered significant. Statistical analysis was performed using GraphPad prism 9.5.0 version (San Diego, CA, USA). For comparative analysis with the LCE tool, Welch’s two-sample *t*-test assuming unequal variance was used to generate the *p*-value. In the resulting box and whisker plot, the lower whisker extends from the lower quartile to the lowest smaller value within a 1.5 interquartile range (IQR), whereas the upper whisker extends from the upper quartile to the highest larger value within a 1.5 IQR. The red solid dot and the value beside it represents the mean [37].

## Figures and Tables

**Table 1:** Circulating S1P levels and S1PR3 tissues expression in male and female ADK and SCC patients.

	Male		Female	
	S1P (Median)	S1PR3 (Median)	S1P (Median)	S1PR3 (Median)
**ADK**	89.83 nM	1.52	70.13 nM	1.5
	[79.81–96.80 range]	[0.63–3.55 range]	[61.98–96.80 range]	[0.55–2.09 range]
**SCC**	81.06 nM	1.99	126 nM	2.21
	[60.31–108.6 range]	[1.158–2.924 range]	[107.7–138.3 range]	[1.041–3.583 range]

## Data Availability

Not applicable.

## Supplementary Materials

The following supporting information can be downloaded at: https://www.mdpi.com/article/10.3390/ijms241310841/s1.
