# Supplementary material for: Sex Differences in Sphingosine-1-Phosphate Levels Are Dependent on Ceramide Synthase 1 and Ceramidase in Lung Physiology and Tumor Conditions
Source: Int J Mol Sci. 2023 Jun 29;24(13):10841. doi: 10.3390/ijms241310841 (PMC10341818; doi:10.3390/ijms241310841)
Supplement: Supplementary file 1 [file ijms-24-10841-s001.zip › ijms-2451352-supplementary.pdf]

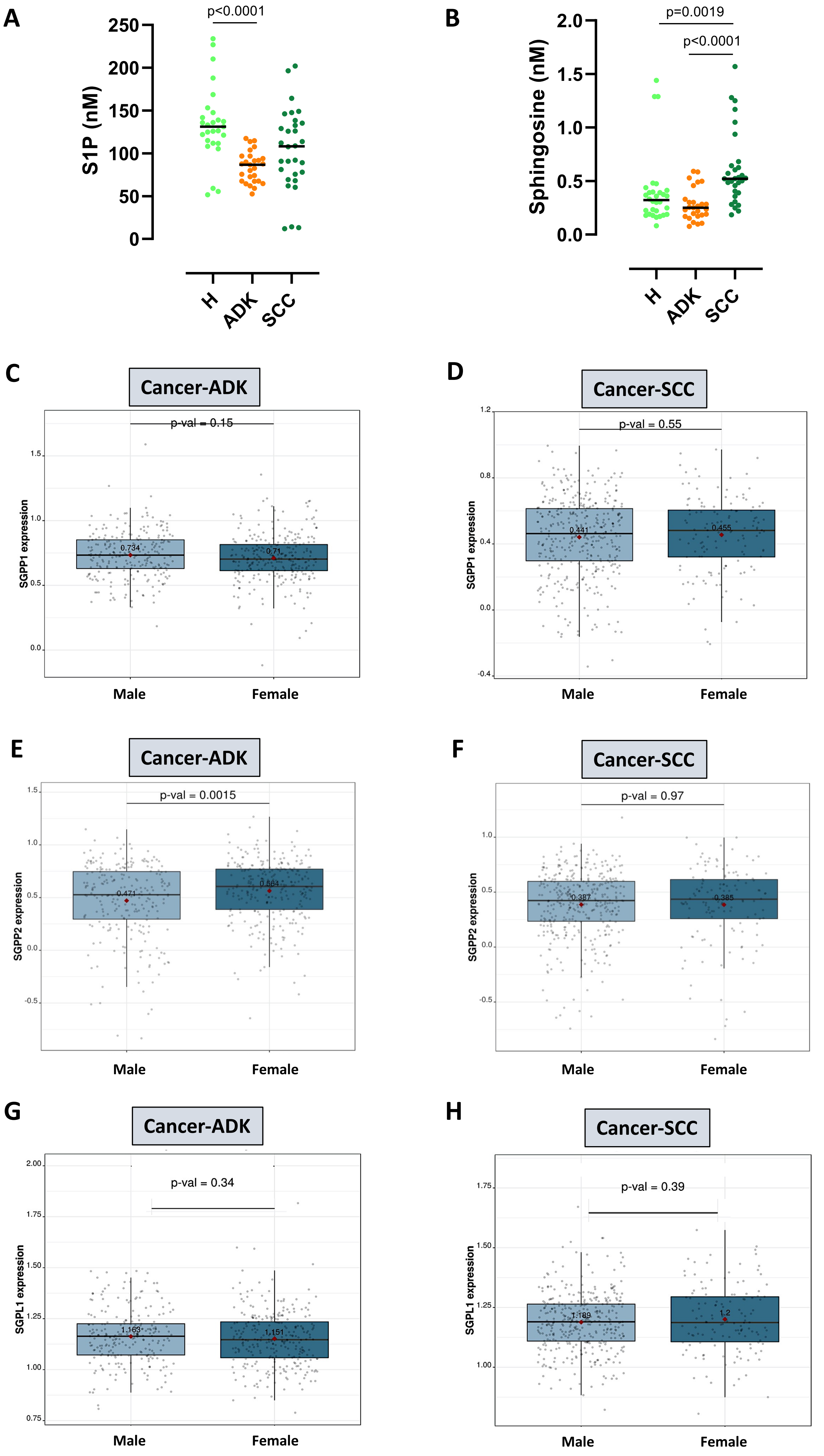

Supplementary Figure S1

Supplementary Figure S1. S1P (**A**) and sphingosine (**B**) quantification in plasma of H (n=27), ADK (n=37) and SCC patients (n=30), by means of LC-MS/MS. Statistical differences were assessed by means of One-way ANOVA followed by Dunn's post hoc test. H: healthy; ADK: lung adenocarcinoma; SCC: lung squamous cell carcinoma. Transcripts quantification of sphingosine-1-phosphate phosphatase 1 and 2 (SGPP1 and SGPP2) in cancerous lung tissues of ADK (**C** and **E**, male=240; female n=277) and SCC (**D** and **F**, male=371; female n=130) patients. Transcripts quantification of sphingosine-1-phosphate lyase 1 (SGPL1) in cancerous lung tissues of ADK (**G**, male=240; female n=277) and SCC (**H**, male=371; female n=130) patients. Data were from RNAseq TCGA_LUAD_2016, for lung adenocarcinoma patients, and TCGA_LUSC_2016 dataset, for lung squamous cell carcinoma patients. A comparative analysis of transcripts with default parameters was performed by means of Lung Cancer Explorer (LCE) tool .

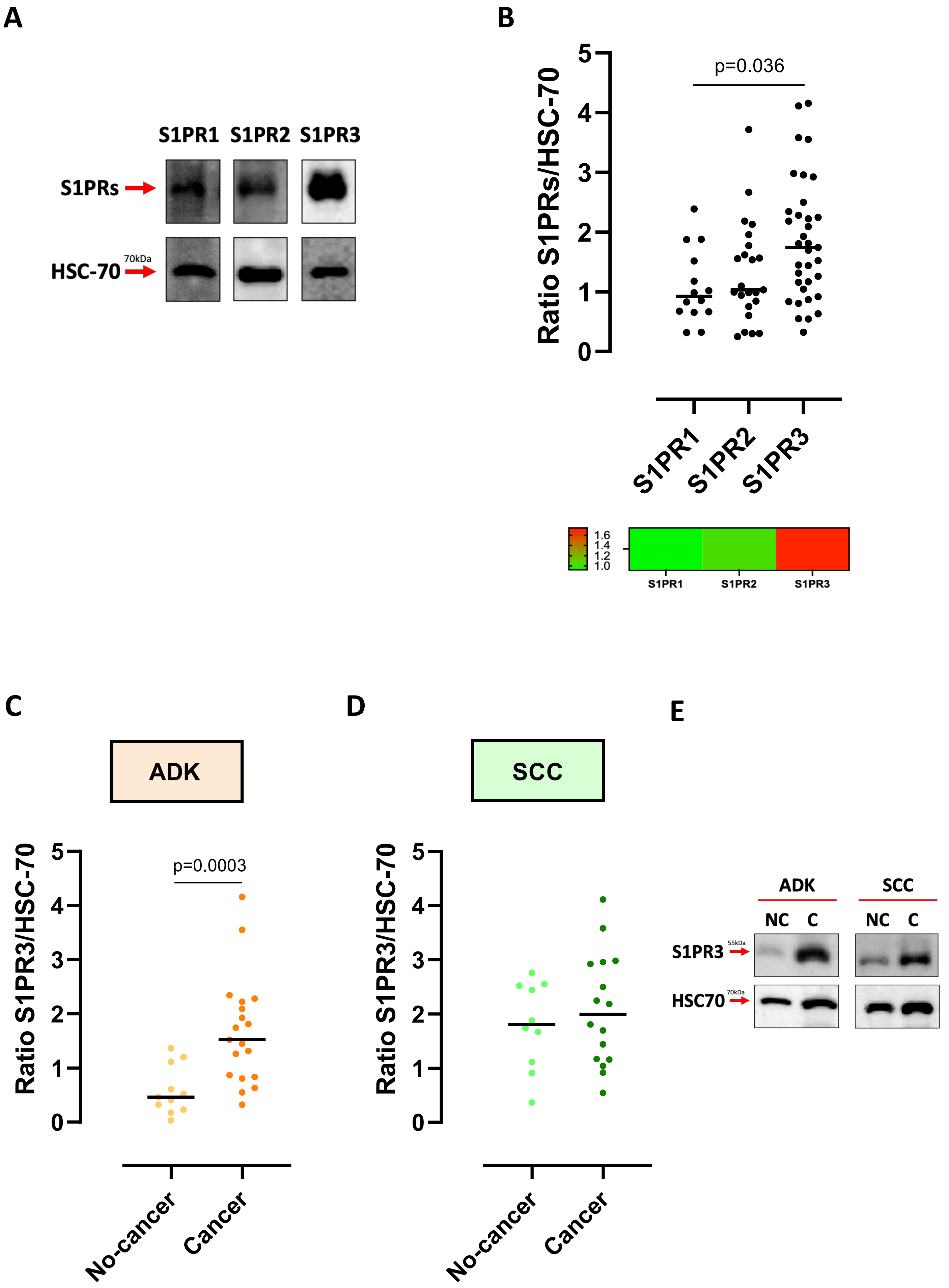

Supplementary Figure S2

Supplementary Figure S2. Expression of S1PRs on lung cancerous tissues. S1PR3 (55kDa; n=35) was over-expressed in lung cancerous tissues, compared to S1PR1 (44kDa; n=14) and S1PR2 (40-50kDa; n=23). HSC70 was used as loading control (**A** and **B)** . Data are represented as scatter dot plots indicating the median. Statistical differences were assessed by means of One-way ANOVA followed by Dunn's post hoc test. Western blotting analysis showed a higher expression of S1PR3 (55kDa) in cancerous lung tissues of ADK patients (Cancer, n=19) compared to non-cancerous lung tissues (No-cancer, n=11) (**C**), while cancerous (Cancer, n=16) and non-cancerous (No-cancer, n=10) lung tissues of SCC patients had similar expression of S1PR3 (**D**). **E)** Representative blot for S1PR3 expression in non-cancerous (NC) and cancerous (C) tissues of ADK and SCC patients. The quantitative analysis was performed by ImageJ software. Data are represented as scatter dot plots indicating the median. Statistical differences were assessed by means of two-tailed Mann-Whitney U test. S1PR1: sphingosine-1-phosphate receptor 1; S1PR2: sphingosine-1-phosphate receptor 2; S1PR3: sphingosine-1-phosphate receptor 3; HSC70: Heat shock cognate protein 70; ADK: lung adenocarcinoma; SCC: lung squamous cell carcinoma.

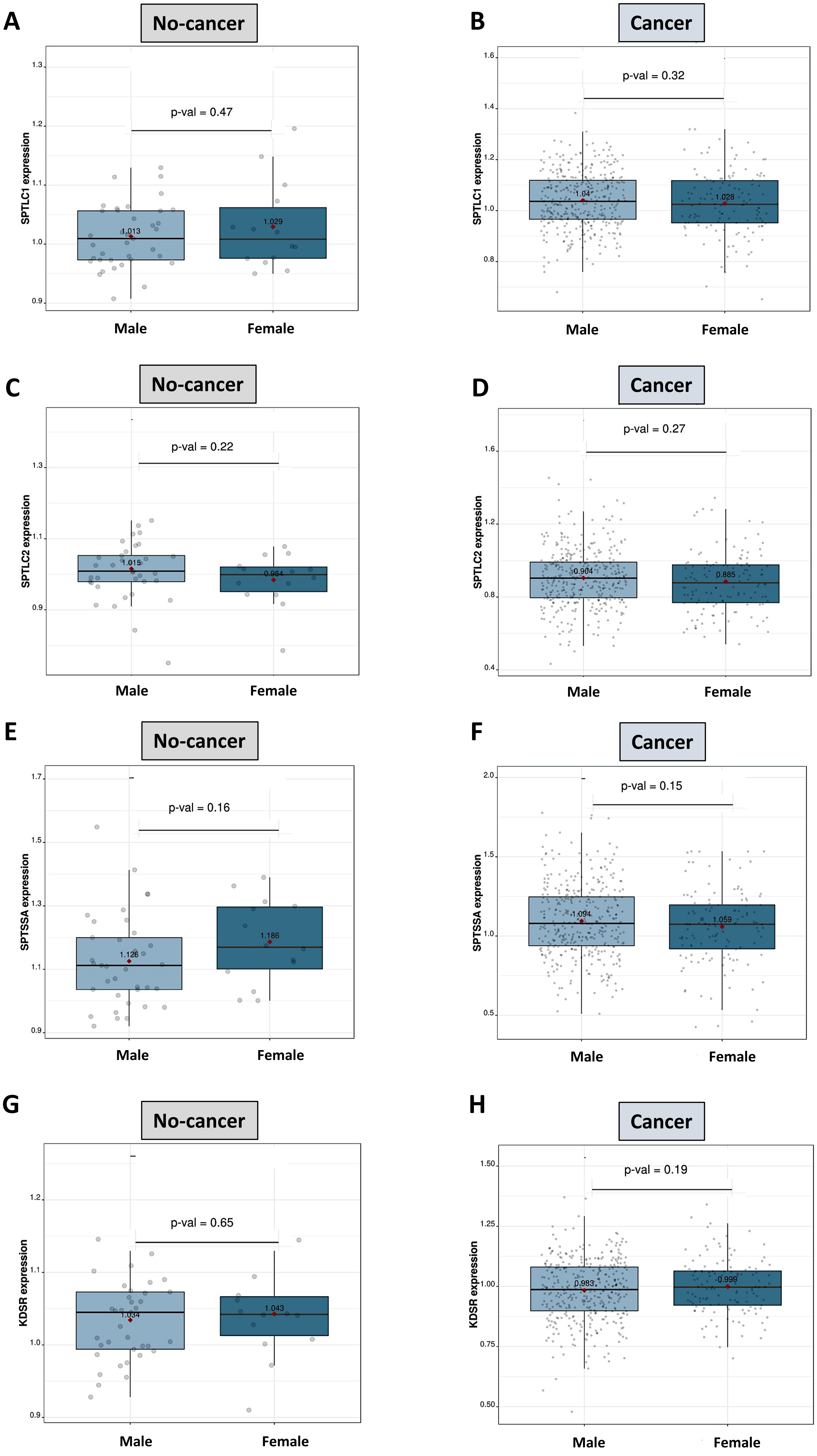

Supplementary Figure S3

Supplementary Figure S3. Expression of S1P metabolic enzymes involved in the first two steps of *de novo* synthesis. Transcripts expression of the S1P *de novo* synthesis enzymes in non-cancerous (No-cancer, male n=37, female n=14) and cancerous (Cancer, male n=371, female n=130) lung tissues of SCC patients from TCGA_LUSC_2016 dataset. Serine palmitoyl transferase (SPT) subunits: Serine palmitoyl transferase long chain base subunit 1 and 2 (SPTLC1 and 2) and serine palmitoyl transferase small subunit A (SPTSSA) (**A-F**); 3-ketodihydrosphingosine reductase (KDSR) (**G-H**). A comparative analysis of transcripts with default parameters was performed by means of Lung Cancer Explorer (LCE) tool.

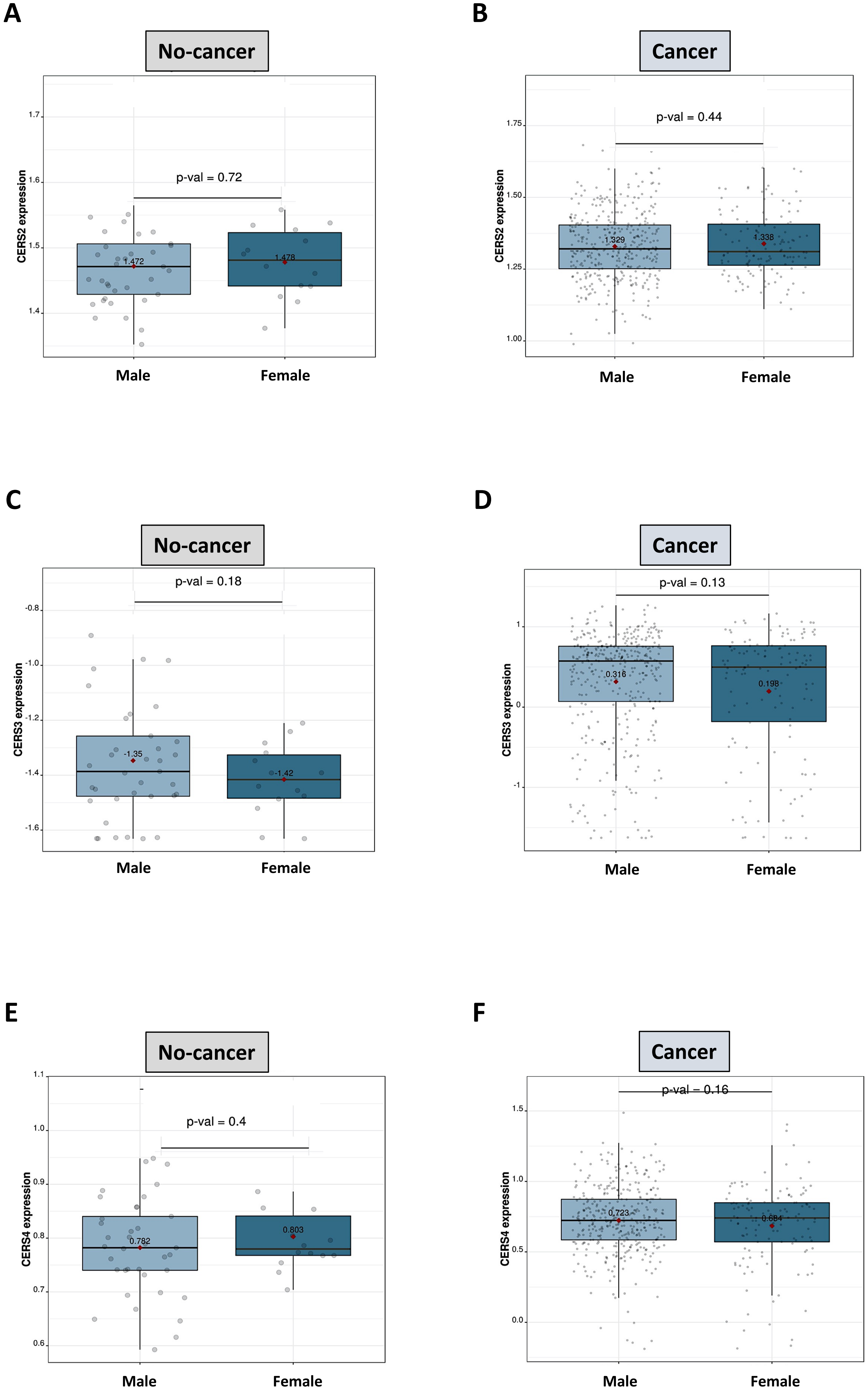

Supplementary Figure S4

Supplementary Figure S4. Expression of S1P metabolic enzymes producing dihydroceramide starting from dihydrosphinganine. Transcripts expression of the S1P *de novo* synthesis enzymes in non-cancerous (No-cancer, **A**, **C** and **E**, male n=37, female n=14) and cancerous (Cancer, **B**, **D** and **F**, male n=371, female n=130) lung tissues of SCC patients from TCGA_LUSC_2016 dataset: ceramide synthase 2 (CERS2, **A** and **B**), 3 (CERS3, **C** and **D**) and 4 (CERS4, **E** and **F**). A comparative analysis of transcripts with default parameters was performed by means of Lung Cancer Explorer (LCE) tool.

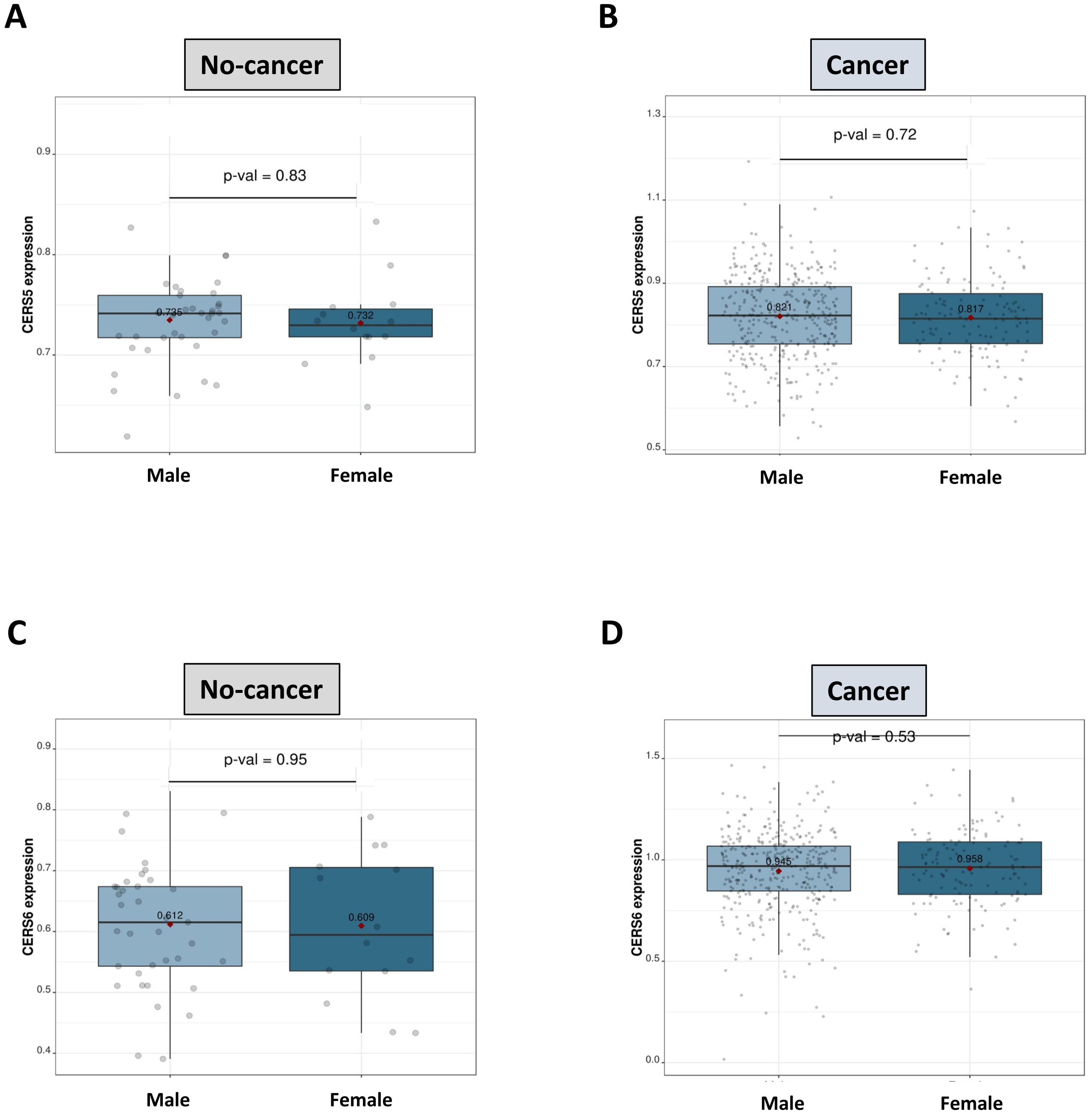

Supplementary Figure S5. Expression of S1P metabolic enzymes producing dihydroceramide starting from dihydrosphinganine. Transcripts expression of the S1P *de novo* synthesis enzymes in non-cancerous (No-cancer, **A** and **C**, male n=37, female n=14) and cancerous (Cancer, **B** and **D**, male n=371, female n=130) lung tissues of SCC patients from TCGA_LUSC_2016 dataset: ceramide synthase 5 (CERS5, **A** and **B**) and 6 (CERS6, **C** and **D**). A comparative analysis of transcripts with default parameters was performed by means of Lung Cancer Explorer (LCE) tool.

Supplementary Figure S5

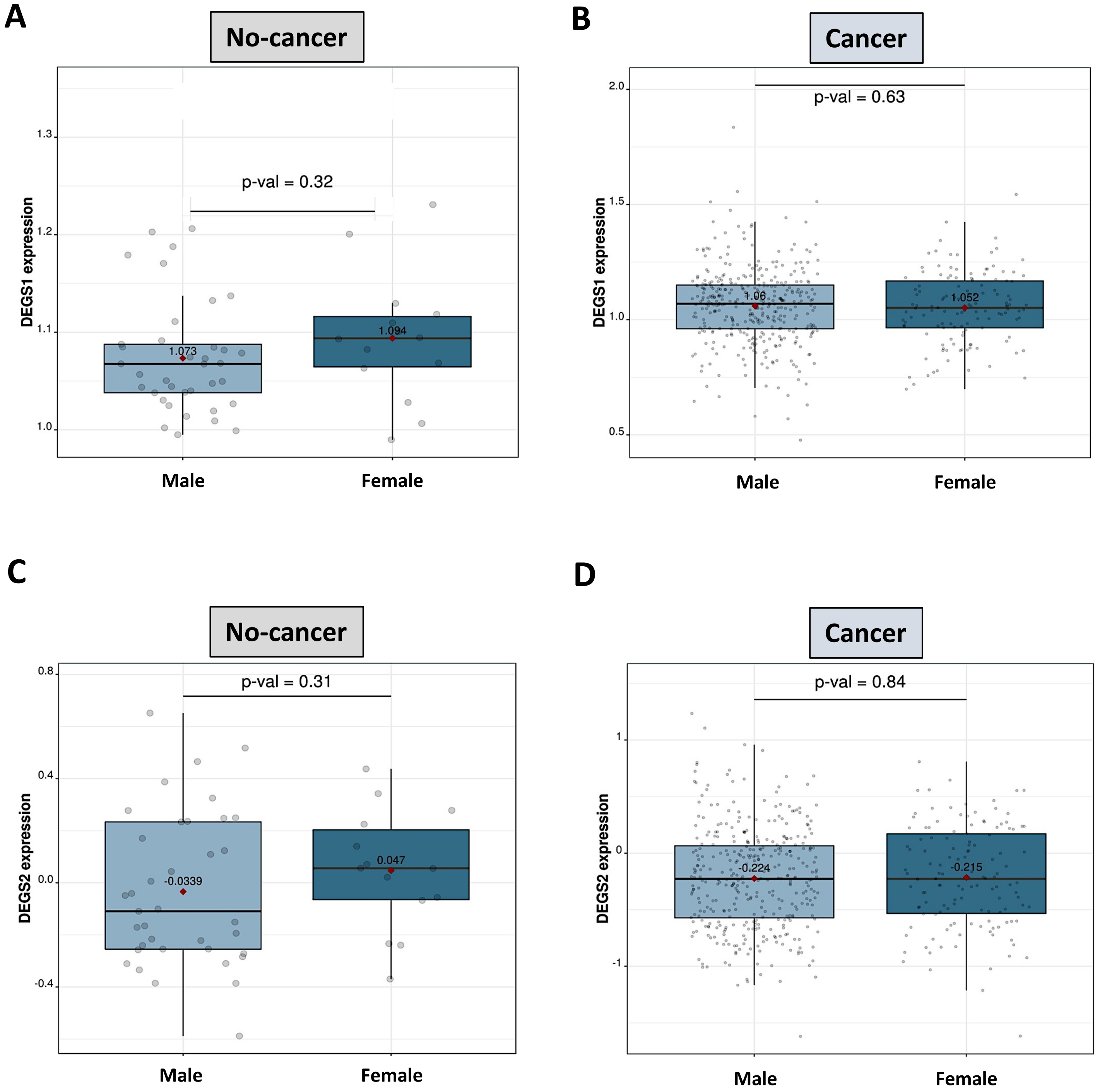

Supplementary Figure S6. Expression of S1P metabolic enzymes producing ceramide starting from dihydroceramide. Transcripts expression of the S1P *de novo* synthesis enzymes in non-cancerous (No-cancer, male n=37, female n=14) and cancerous (Cancer, male n=371, female n=130) lung tissues of SCC patients from TCGA_LUSC_2016 dataset. Dihydroceramide desaturase 1 (DEGS1, **A** and **B**) and 2 (DEGS2, **C** and **D**). A comparative analysis of transcripts with default parameters was performed by means of Lung Cancer Explorer (LCE) tool.

Supplementary Figure S6

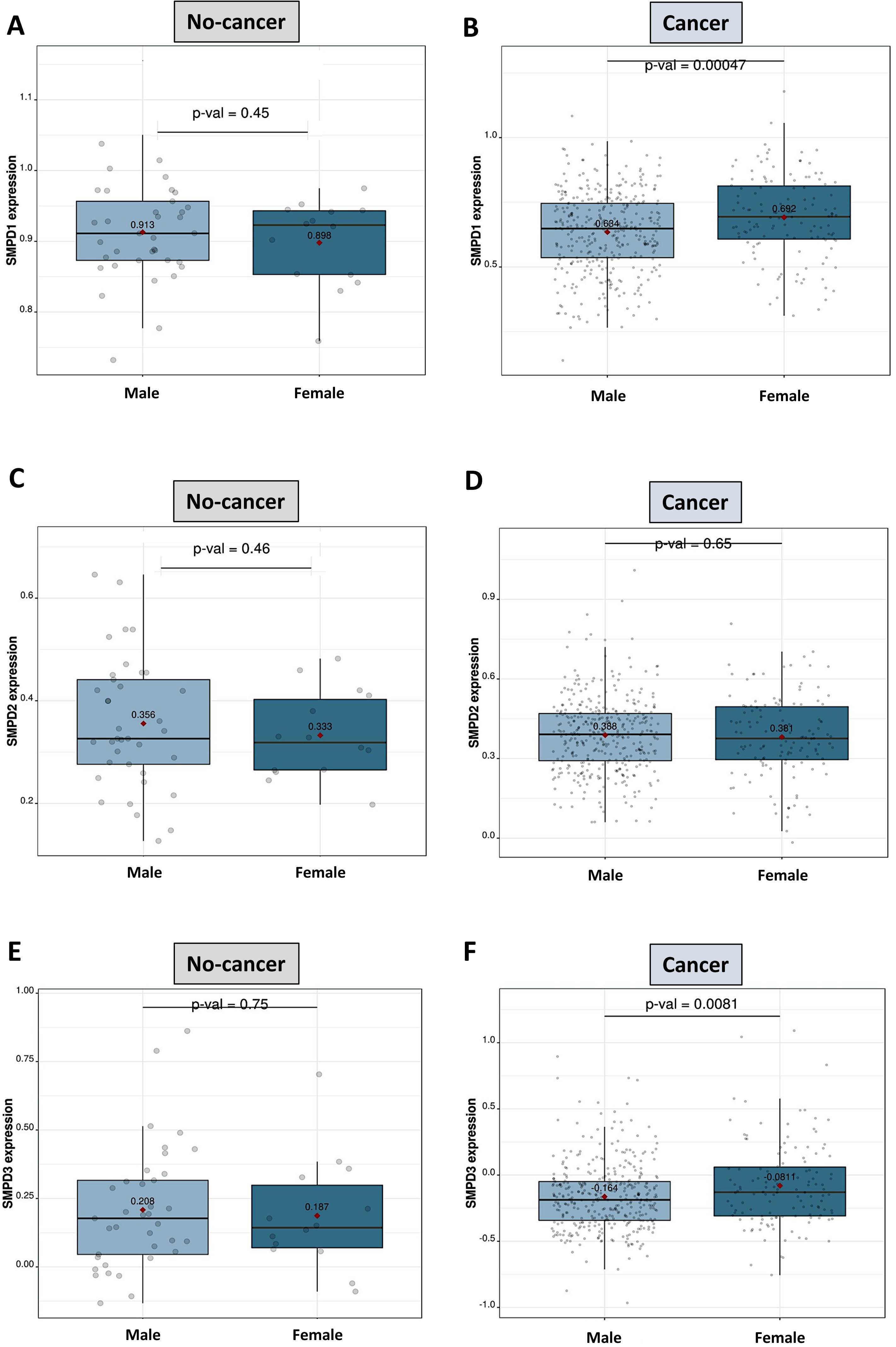

Supplementary Figure S7

Supplementary Figure S7. Expression of S1P metabolic enzymes involved in the sphingomyelin hydrolysis. Transcripts expression of the sphingomyelin-dependent S1P synthesis enzymes in non-cancerous (No-cancer, male n=37, female n=14) and cancerous (Cancer, male n=371, female n=130) lung tissues of SCC patients from TCGA_LUSC_2016 dataset. Sphingomyelinase 1 (SMPD1, **A** and **B**), 2 (SMPD2, **C** and **D**), and 3 (SMPD3, **E** and **F**). A comparative analysis of transcripts with default parameters was performed by means of Lung Cancer Explorer (LCE) tool.

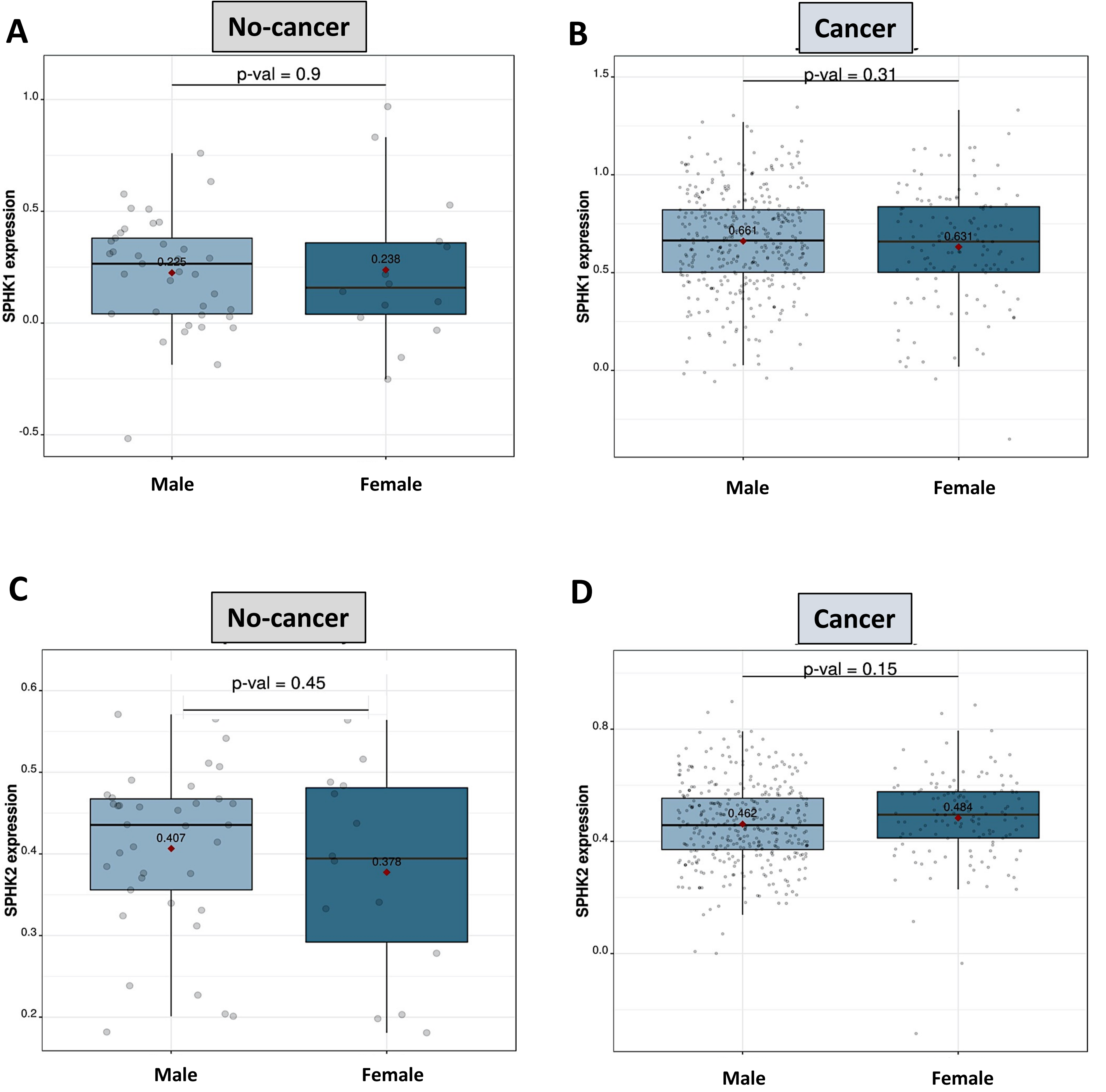

Supplementary Figure S8. Expression of S1P metabolic enzymes. Transcripts expression of the sphingosine kinase 1 (SPHK1) (**A** and **B**), sphingosine kinase 2 (SPHK2) (**C** and **D**) in non-cancerous (No-cancer, male n=37, female n=14) and cancerous (Cancer, male n=371, female n=130) lung tissues of male and female SCC patients from TCGA_LUSC_2016 dataset. A comparative analysis of transcripts with default parameters was performed by means of Lung Cancer Explorer (LCE) tool.

Supplementary Table S1. Healthy subjects informations. H: healthy.

Healthy Subjects	Sex	Age (years)
**H1**	Female	68
**H2**	Female	75
**H3**	Female	69
**H4**	Female	76
**H5**	Female	42
**H6**	Female	60
**H7**	Female	67
**H8**	Female	82
**H9**	Female	73
**H10**	Female	77
**H11**	Female	41
**H12**	Female	35
**H13**	Male	53
**H14**	Male	59
**H15**	Male	66
**H16**	Male	67
**H17**	Male	55
**H18**	Male	33
**H19**	Male	49
**H20**	Male	78
**H21**	Male	63
**H22**	Male	61
**H23**	Male	59
**H24**	Male	59
**H25**	Male	46
**H26**	Male	49
**H27**	Male	53

Supplementary Table S2. Non-small cell lung cancer (NSCLC) patients informations. LC: lung cancer; ADK: lung adenocarcinoma; SCC: lung squamous cell carcinoma.

NSCLC patients	Sex	Histotype	Age (years)
**LC1**	Female	ADK	72
**LC2**	Female	ADK	67
**LC3**	Female	ADK	73
**LC4**	Female	ADK	59
**LC5**	Female	ADK	68
**LC6**	Female	ADK	57
**LC7**	Female	ADK	54
**LC8**	Female	ADK	63
**LC9**	Female	ADK	44
**LC10**	Female	ADK	62
**LC11**	Female	ADK	62
**LC12**	Female	ADK	75
**LC13**	Male	ADK	66
**LC14**	Male	ADK	71
**LC15**	Male	ADK	42
**LC16**	Male	ADK	80
**LC17**	Male	ADK	83
**LC18**	Male	ADK	69
**LC19**	Male	ADK	66
**LC20**	Male	ADK	79
**LC21**	Male	ADK	66
**LC22**	Male	ADK	75
**LC23**	Male	ADK	56
**LC24**	Male	ADK	41
**LC25**	Male	ADK	49
**LC26**	Male	ADK	59
**LC27**	Male	ADK	58
**LC28**	Female	SCC	67
**LC29**	Female	SCC	63
**LC30**	Female	SCC	68
**LC31**	Female	SCC	40
**LC32**	Female	SCC	55
**LC33**	Female	SCC	77
**LC34**	Female	SCC	35
**LC35**	Female	SCC	49
**LC36**	Female	SCC	69
**LC37**	Female	SCC	35
**LC38**	Female	SCC	59
**LC38**	Female	SCC	82
**LC40**	Female	SCC	27
**LC41**	Female	SCC	70
**LC42**	Female	SCC	48
**LC43**	Male	SCC	45
**LC44**	Male	SCC	54
**LC45**	Male	SCC	57
**LC46**	Male	SCC	52
**LC47**	Male	SCC	69
**LC48**	Male	SCC	42
**LC49**	Male	SCC	53
**LC50**	Male	SCC	49
**LC51**	Male	SCC	40
**LC52**	Male	SCC	50
**LC53**	Male	SCC	43
**LC54**	Male	SCC	50
**LC55**	Male	SCC	55
**LC56**	Male	SCC	35
**LC57**	Male	SCC	72
